# A sub-group evaluation of the multi-month dispensing strategy for differentiated HIV care: is personalization of care guidelines warranted in Haiti?

**DOI:** 10.1186/s12913-022-07475-8

**Published:** 2022-01-16

**Authors:** Canada Parrish, Anirban Basu, Paul Fishman, Jean Baptiste Koama, Ermane Robin, Kesner Francois, Jean Guy Honoré, Joëlle Deas Van Onacker, Nancy Puttkamme

**Affiliations:** 1 University of Washington, Magnuson Health Sciences Building, 1705 NE Pacific Street, Seattle, WA 98195 USA; 2 Centers for Disease Control and Prevention, Port-au-Prince, Haiti; 3 Programme National de Lutte contre le VIH/SIDA (PNLS), Ministère de la Santé Publique et de la Population (MSPP), Port-au-Prince, Haiti; 4 Centre Haïtien pour le Renforcement du Système de Santé (CHARESS), Port-au-Prince, Haiti

**Keywords:** HIV service delivery, Haiti, Care guidelines

## Abstract

**Background:**

Differentiated care strategies are rapidly becoming the norm for HIV care delivery globally. Building upon an interest in tailoring antiretroviral therapy (ART) delivery for client-centered needs, the Ministry of Health and Population in Haiti formally endorsed multiple-month dispenses (MMD) in the 2016 national ART guidelines This study explores heterogeneity in retention in care with MMD for specific Haitian populations living with HIV and evaluates if a targeted algorithm for optimal ART prescription intervals is warranted in Haiti.

**Methods:**

This study included ART-naïve individuals who started ART on or after January 1st, 2017 in Haiti. To identify subgroups in which to explore heterogeneity of retention, we implemented a double-lasso regression method to determine which individual characteristics would define the subgroups. Characteristics evaluated for potential subgroup definition included: sex, age category, WHO clinical stage, and body mass index category. We employed instrumental variable models to estimate the causal effect of increasing ART dispensing length on ART retention, by client subgroup. The outcome of interest was retention in care after one year in treatment. We then estimated the marginal effect of a 30-day increase to ART dispensing length to retention in care for each of these subgroups.

**Results:**

There was evidence for heterogeneity in the effect of extending ART dispensing intervals on retention by WHO clinical stage. We observed significant improvements to retention in care at one year with a 30-day increase in ART dispense length for all subgroups defined by WHO clinical stages 1-4. The effects ranged from a 14.7% increase (95% CI: 12.4-17.0) to the likelihood of retention for people with HIV in WHO stage 1 to a 21.6% increase (95% CI: 18.7-24.5) to the likelihood of retention for those in WHO stage 3.

**Conclusions:**

All the subgroups defined by WHO clinical stage experienced a benefit of extending ART intervals to retention in care at one year. Though the effect did differ slightly by WHO stage, the effects went in the same direction and were of similar magnitude. Therefore, a standardized recommendation for MMD among those living with HIV and new on ART is appropriate for Haiti treatment guidelines.

## Background

Differentiated care strategies are rapidly becoming the norm for HIV care delivery globally [1, 2]. Eligibility criteria for differentiated HIV care have expanded and there is increased focus on how to best implement treatment and support services for all people with HIV (PWH) [1]. One differentiated care approach has been to increase (ART) dispensing lengths to several month intervals to ease the demands on individuals’ time in obtaining prescription refills [1, 3]. This approach also benefits healthcare systems that are managing care for an increasing number of PWH by reducing the human resources required to sustain care delivery. Studies have demonstrated that longer ART dispensing intervals may be clinically beneficial for patient outcomes; longer ART intervals are associated with increased probability of retention in care and viral suppression [4, 5].

Building upon an interest in further reducing barriers to ART retention and prior experience in informally implementing Multiple-Month Dispenses (MMD) in some clinics, the Ministry of Health in Haiti formally endorsed MMD for PWH who were “clinically and virologically stable,” defined as being on ART for at least 6 months with no emergent opportunistic infections or evidence of a detectable viral load, in the November 2016 national ART guidelines [6]. The guidelines, however, do not state precise prescription lengths or specify tailored recommendations based on patient characteristics. Previous research estimated that increasing ART dispensing length by 30 days would have improved the likelihood of retention by up to 23% for those with ART dispenses of between 20 and 50 days, and that all PWH with dispenses of up to 130 days would have benefited from longer intervals [7]. The causal estimates from that research represented population-averaged intervention effects; the current study aims to address whether the effect on retention differed by various subgroups.

Subgroup analyses are common in the medical literature; they build upon existing evidence by adding a focus on particular groups of interest, potentially leading to more nuanced, appropriate policies than population wide-recommendations [8]. Treatment or intervention effects may differ by individuals and across different populations*,* this is referred to as heterogeneity [9, 10]. There are important clinical implications for characterizing this heterogeneity for certain treatment guidelines. Clinicians may find that information from subgroup analyses can be more interpretable than deriving information for an individual patient using data from a population-based model [11, 12]. Evidence informed policies for specific patient groups can alleviate some of the clinical decision-making burden providers face when deciding between a range of treatment options for a given patient [13]. In many low and middle-income countries (LMICs), a low number of doctors per capita and task sharing with lower-level cadres of health workers [14, 15] make protocolized guidelines desirable for consistent, evidence-informed care pathways.

Subgroup analyses are made possible by the availability of data from data-rich systems with large amounts of information, such as regional or national electronic health records. Clinical trials are not often powered for subgroup analyses which reduce the analytic sample size [16, 17], so subgroup analyses from observational datasets represent important contributions to the literature about the effect of specific clinical strategies or treatment protocols. Finally, subgroup analyses are helpful to prepare for and manage specific patient populations where medication supply chain needs, provider time demands, and facility procedures may differ for different treatment options optimized to certain patient groups.

This study explores heterogeneity in retention in care with increasing ART dispensing lengths for specific Haitian HIV patient subgroups and evaluates if a targeted algorithm for optimal ART dispensing intervals is preferable to a uniform, population-based strategy in Haiti. This information will be useful for clinician decision-making and for policy makers in defining whether sub-group recommendations for ART dispensation interval are warranted within Haiti’s national ART guidelines.

## Methods

This study used the iSanté HIV-specific electronic health record (EHR) from Haiti. iSanté is a networked system of longitudinal clinical encounter data used in more than 100 Haitian health facilities [18]. Approximately three-quarters of all facilities that offer HIV care services are represented in the database and iSanté contains the medical records for approximately 63% of people enrolled in HIV-support services in the country [19]. iSanté includes information regarding demographics, laboratory history/results, diagnosis history, treatment history, and pharmacy records, as well as data fields for counseling and referrals received [20, 21]. Our sample included ART-naïve PWH enrolled in iSanté who started ART on or after January 1st, 2017 and could have been followed for 13-months (to assess 1-year retention with a 30-day grace period) after their initial ART dispense.

### Variables

The primary exposure was ART dispense length in days, calculated by the average dispensing length over the first 6 months of treatment, as defined in our prior research [7]. Initial dispense lengths tend to be short, several days to one week, so that clinicians can closely monitor PWH for ART toxicity, side effects, and patient tolerance to their ART regimens [22]. We chose to classify the exposure in which manner based on the fact there is high attrition in early care [18] and high within-person variability in ART dispense lengths during the first months of treatment.

The outcome was a binary measure of retention (retained vs. not retained) in care 12 months after starting ART. This measure was defined by the collection of an ART prescription within 30 days of the scheduled ART pickup after one year in treatment; the Ministry of Health in Haiti definition of an “active” ART client.

Individual characteristics of sex, age, World Health Organization (WHO) clinical stage, and Body Mass Index (BMI) category at ART initiation were included in the models for determining potential subgroups with heterogeneity in the effect of extending ART dispending intervals and estimating the effect of MMD for those subgroups. WHO Stage is a proxy for HIV disease severity based on clinical characteristics such as the presence of opportunistic infections [23]. To adjust for other variables which can influence treatment adherence in our outcome model, we added an indicator for whether the individual had received appropriate, guideline-informed TB management at ART initiation. Appropriate TB management was defined as being treated for active TB or receiving Isoniazid (INH) for TB prophylaxis. In addition, we included facility-level categorical variables for facility ownership (public, private, and mixed) and facility network to account for differences in clinical practices and guideline implementation. The WHO stage and BMI category variables included a “missing” indicator if there was insufficient evidence in the medical record to ascertain these characteristics.

The key exposure, outcome, and adjustment variables are described in greater detail in our previous work estimating the population-averaged, causal effect of extending ART dispensing intervals on ART retention in Haiti [7].

### Data analysis

To identify subgroups in which to explore heterogeneity, we implemented a double-lasso regression method [24, 25] for variable selection to determine which individual characteristics would define the subgroups. The lasso (least absolute shrinkage and selection operator) is a type of regression method designed to improve the prediction accuracy and interpretability of regression models by selecting a subset of the available covariates that exhibit the strongest effects [24]. We first used a data-driven penalization lasso approach for predicting ART dispensing length in days and then a regularized logistic regression model for the outcome of retention. The first stage model included all possible interactions between the instrument with individual characteristics (sex, age, WHO stage, and BMI) and second stage models included interactions with the same individual-level variables and ART dispense length. Main effects of the individual characteristics, TB management, and facility-level variables were not penalized in either model. Interactions that were selected by the lasso algorithm in either model were included in the IV analysis.

We used an instrumental variable (IV) analysis using a 2-stage residual inclusion (2SRI) approach for non-linear models [9, 26] to estimate the causal effect of increasing ART dispensing length on ART retention [7], by subgroup. The instrument used was the rolling 6-month mean facility-level ART dispensing interval for new ART recipients. The validity and strength of the chosen instrument were assessed for the first stage model. We assessed the goodness-of-fit of the second stage outcome models.

All included interaction terms were tested for significance jointly across all levels of the variable after the second stage model in the IV analysis. Significant interaction terms indicated characteristics in which treatment heterogeneity existed for this population. Individual characteristics that were in statistically significant interaction terms then defined the subgroups of interest. We estimated the marginal effect of a 30-day increase to ART dispensing interval to retention in care for each of these subgroups using the margins post-estimation command following the second stage IV model and compared these marginal effects across groups. Bootstrapping with 1000 iterations was conducted for valid standard errors with the modeling approach.

We conducted a secondary analysis among the subgroups identified in our primary analysis to test if the effect of extending ART dispenses by 30 days differed across different observed ART dispensing intervals. We estimated the effects of increasing ART dispensing length for subgroups among three observed ART intervals categories: less than 60 days, 60-90 days, and more than 90 days in length. These categories were informed by our previous work demonstrating the population-averaged effect of increases to ART dispensing intervals differed across these three categories [7].

### Ethical review

The study protocol was reviewed and approved by University of Washington’s Human Subjects Division and the Haiti Ministry of Health’s National Bioethics Committee, and was also reviewed in accordance with the US Centers for Disease Control and Prevention (CDC) human research protection procedures guidelines. All methods performed were in accordance with the institutional guidelines. All three ethics bodies provided a consent waiver for the secondary use of de-identified individual-level data for this study and none of the researchers had contact with the study participants.

## Results

The demographic and clinical characteristics of the 21,880 ART naïve PWH in the study population included in this analysis are described elsewhere [7].

Interactions with all the individual-level variables were selected via the double-Lasso selection process and were included in the following IV analysis in both the first and second stage models. Individual level covariates were balanced between the instrument levels when the instrument was divided at the median. The instrument was strong in the first stage models; highly predictive of individual ART dispense length. The second stage model had satisfactory performance in our employed goodness-of-fit tests.

**Fig. 1:**
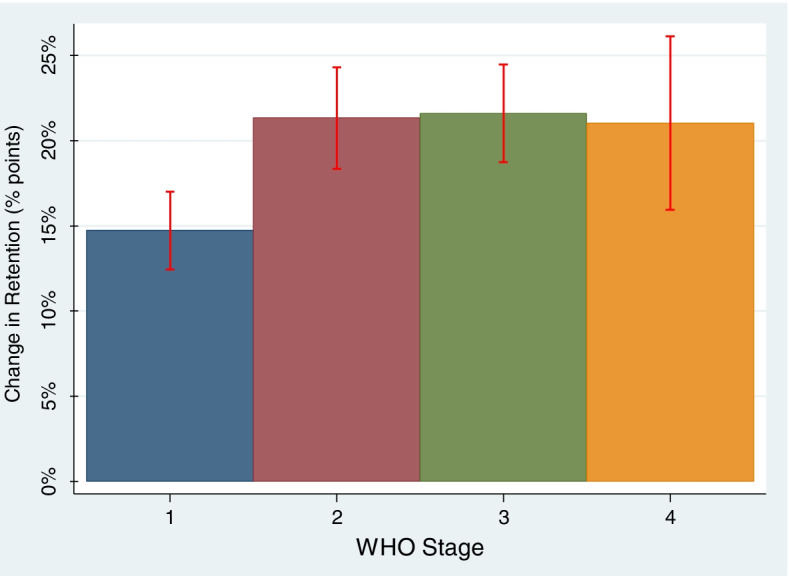
Effects of a 30-day Increase to ART Dispense Length on Retention in Care for WHO Stage subgroups

In the adjusted outcome model, only the interactions between ART dispense and WHO stage were statistically significant. There was evidence for heterogeneity in the effect of extending ART dispensing intervals on retention in care among WHO stages. This selected characteristic of WHO stage, as operationalized in our analyses, then defined our 4 unique ART recipient subgroups. Within the 4 subgroups, we observed improvements to the likelihood of retention in care at 1 year with increased ART dispensing lengths for all subgroups with statistically significant marginal effects (Fig. 1). The effects ranged from a 14.7% increase (95% CI: 12.4-17.0) in the likelihood of retention with a 30-day extension of the ART dispensing interval for PWH in WHO stage 1 to a 21.6% increase (95% CI: 18.7-24.5) in the likelihood of retention for those in WHO stage 3. The increases in likelihood of retention for PWH in WHO Stages 2 and 3 were significantly greater than the increases for PWH in WHO Stage 1. The changes to retention for PWH in WHO Stage 4 were not statistically significantly different than the increases in retention estimated for those in WHO Stages 1-3. All changes to retention were positive (*increased* retention) and of comparable magnitude in the WHO stage subgroups.

**Fig. 2:**
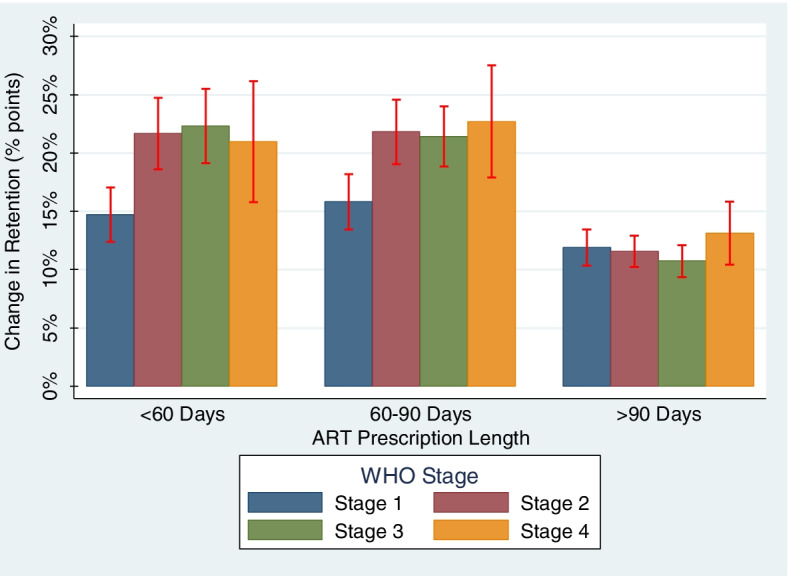
Effects of a 30-day Increase to ART Dispense Length on Retention in Care across 3 ART Prescription Length Categories

We conducted our secondary analysis among the WHO stage subgroups and the prespecified ART dispense length categories. Individuals in all WHO stage subgroups were estimated to have statistically significant improvements to retention with increased ART dispensing length across all three ART length categories (Fig. 2). Effects ranged from 10.7% (95%CI: 9.1-12.3) for WHO stage 3 PWH with ART dispenses more than 90 days in length to 22.7% (95%CI: 18.0-27.4) for 60-90 day dispensing intervals in WHO stage 4. The maximum benefit of extending dispensing intervals was observed for those currently on 60-90 day dispenses. For ART dispensing length categories of less than 60 days and 60-90 days, PWH in WHO stages 2 and 3 had significantly larger increases in the likelihood of retention compared to those in WHO stage 1. For those currently on dispensing intervals of more than 90 days, there was no difference to the increases to retention across the WHO stage groups. Overall, the benefits to retention were smallest among the subgroups with dispensing intervals in that ART length category.

## Discussion

Statistically significant improvements to the likelihood of retention in care with 30-day increases in ART dispensing length were demonstrated for all groups and effects were of similar magnitude across the subgroups defined by WHO clinical stage. Although heterogeneity in the effect of lengthening dispensing interval was observed, the improvements to retention were not different enough to warrant further exploration of a targeted ART dispense length policy based on individual characteristics. One set of prescribing guidelines appears to be sufficient to increase retention in care for all Haitian ART recipients. However, it is possible that those in later WHO clinical stages may need more contact with the healthcare system, especially in the early treatment stages, to manage the other conditions (such as opportunistic infections) which characterize these later stages. As noted in our secondary analysis, the overlapping confidence intervals for the WHO stage subgroups suggest similar trends across PWH groups and observed ART dispense lengths up to 90 days. Although we did not observe significant, differential effects of MMD on retention in care across groups defined by age, sex, and BMI, clinicians will likely use these factors to tailor ART treatment regimens and care trajectories for individuals for reasons other than retention, such as care preferences or other competing health priorities.

This study had several limitations, some of which are related to our data source and methodological approach in estimating the causal effect of increasing ART dispense length on retention (IV analysis) and are described in previous work [7]. Lasso regression selects the variables with the strongest effects on the outcome [24, 27], not necessarily the variables most commonly used to consider treatment options in clinical practice. Variable selection by this process may not be intuitive for clinicians. To note, we did not identify sex, age, or BMI as variables to evaluate for heterogeneity in the intervention effect. The variables in which we identified heterogeneity in the intervention effect are highly dependent on this specific Haitian population. Populations with different distributions of individual demographic and clinical characteristics would likely see different subgroups emerge from a similar analytic approach; targeted policies around optimal ART prescription length based on subgroup defining characteristics could be warranted in other settings. Additionally, our EHR database does not reliably capture data to identify men who have sex with men, commercial sex workers, and people who inject drugs; these are often critical populations to consider when optimizing differentiated care strategies. Not characterizing this information in our study sample may limit the ability to generalize our findings to these specific populations or to settings in which the HIV epidemic is concentrated in these populations. Finally, we were unable to precisely estimate the effect of increasing ART dispenses within the defined subgroups at longer ART dispense lengths (longer than 4 months as suggested by our previous analyses) due to limitations in sample size.

However, our approach capitalized on a large population of PWH for most of the other subgroups, our study database contains medical records for a large proportion of Haitians seeking HIV-services, and was able to provide insight into whether further refinement of ART guidelines for Haiti was justifiable. There is a need to balance optimizing individual or subgroup outcomes with ease in guideline and policy implementation and monitoring. A general, uniform policy relating to clinical guidelines appears to be suitable for Haiti. The iSanté EHR allows for the consideration of individual characteristics linked to specific health outcomes and this availability of data allows analysts to run sub-group analyses in order to shape guidelines; however, in many settings, the receipt of monitoring information for analysts is most common in the aggregate [28–30]. Additionally, a general guideline of one ART interval length recommendation for all PHW lends well to economic evaluations at the national level [31, 32], where the subgroup defining characteristics may be hard to quantify for an entire population. This general strategy of one ART interval length guideline for all ART recipients could be desirable for other countries that wish to adopt extended ART intervals as a part of their national strategic plan to manage HIV.

These results also provide salient information for care delivery systems in the context of the COVID-19 pandemic and the health system challenges it has engendered. The COVID-19 pandemic has threatened the ART medication supply for over 70 countries due to severely reduced land and air transport services [33]. If medication supplies are limited, our analyses may suggest prioritizing longer ART intervals for groups that would likely experience the most clinical benefit from extended dispensing intervals, such as those in more severe HIV, provided that these individuals receive the appropriate care for the conditions causing the severe morbidity, as antiretrovirals are only one dimension of care for PWH. Extended dispense intervals also helps to ensure that individuals do not run out of medications, even if scheduled visits or check-ins are cancelled or they cannot reach the clinic for the scheduled appointment.

Future research regarding subgroup analysis may be used to characterize the effect of extending ART dispensing intervals among critical populations, such as men who have sex with men, commercial sex workers, and people who inject drugs, to achieve more ambitious 95-95-95 targets for 2030 [34]. These populations are often disproportionally burdened by HIV and frequently experience social and structural barriers to care that hinder ART initiation and ART retention [35, 36]. The effect of extending ART intervals among HIV risk category groups represents area of future research to explore in Haiti. Although iSanté does capture information on transmission risk category, the completeness of this data field can be highly variable and subject to social desirability bias. However, other health information systems may capture this information more consistently and could evaluate the effect of extended ART dispensing intervals on these populations specifically.

## Conclusions

All of the subgroups defined by WHO clinical stage experienced a benefit of extending ART dispensing intervals by 30 days to retention in care at 1 year after ART initiation. Although there was some heterogeneity in WHO staging impacts of a 30-day increase in ART dispensing, the effects went in the same direction and were of similar magnitude. Differentiated prescribing guidelines do not appear to be warranted. Therefore, one, standardized recommendation for ART dispensing length for new ART clients is appropriate for Haiti treatment guidelines. Based on our previous population-based analysis, starting new ART recipients on ART intervals of 3-4 months in length [7], after the possibility of medication toxicity has been ruled out, should be appropriate for most PWH groups. Our results are additionally consistent with our previous analysis in that those already on longer ART intervals may not experience as much of a benefit to retention in further extending dispensing interval as those on shorter dispense lengths.

## Data Availability

Data used in this analysis will not be made available due to protections of medical records data and existing data sharing agreements. Inquiries about the data may be made to the corresponding author.

## Abbreviations

ART: Antiretroviral therapy
BMI: Body mass index
IV: Instrumental variable
MMD: Multiple-month dispenses
PWH: People with HIV
