# Correction to: A sub-group evaluation of the multi-month dispensing strategy for differentiated HIV care: is personalization of care guidelines warranted in Haiti?

**DOI:** 10.1186/s12913-022-07534-0

**Published:** 2022-01-29

**Authors:** Canada Parrish, Anirban Basu, Paul Fishman, Jean Baptiste Koama, Ermane Robin, Kesner Francois, Jean Guy Honoré, Joëlle Deas Van Onacker, Nancy Puttkammer

**Affiliations:** 1 University of Washington, Magnuson Health Sciences Building, 1705 NE Pacifc Street, Seattle, WA 98195 USA; 2 Centers for Disease Control and Prevention, Port-au-Prince, Haiti; 3 Programme National de Lutte contre le VIH/ SIDA (PNLS), Ministère de la Santé Publique et de la Population (MSPP), Port-au-Prince, Haiti; 4 Centre Haïtien pour le Renforcement du Système de Santé (CHARESS), Port-au-Prince, Haiti

**Correction to: BMC Health Serv Res 22, 80 (2022)**

**https://doi.org/10.1186/s12913-022-07475-8**

Following publication of this article [1], the authors identified an error in the author name of Nancy Puttkammer.

The incorrect author name is: Nancy Puttkamme.

The correct author names is: Nancy Puttkammer.

The author group has been updated above and the original article [1] has been corrected.
